# *Siraitia grosvenorii* Residual Extract Inhibits Inflammation in RAW264.7 Macrophages and Attenuates Osteoarthritis Progression in a Rat Model

**DOI:** 10.3390/nu15061417

**Published:** 2023-03-15

**Authors:** Yun Mi Lee, Misun Kim, Heung Joo Yuk, Seung-Hyung Kim, Dong-Seon Kim

**Affiliations:** 1 KM Science Research Division, Korea Institute of Oriental Medicine, Daejeon 34054, Republic of Korea; candykong@kiom.re.kr (Y.M.L.);; 2 Institute of Traditional Medicine and Bioscience, Daejeon University, Daejeon 34520, Republic of Korea

**Keywords:** *Siraitia grosvenorii* residual extract, inflammation, extracellular matrix (ECM), osteoarthritis

## Abstract

Osteoarthritis (OA) is a degenerative joint disease characterised by cartilage degeneration and chondrocyte inflammation. We investigated the anti-inflammatory effects of the *Siraitia grosvenorii* residual extract (SGRE) in lipopolysaccharide (LPS)-induced RAW264.7 macrophages in vitro and its anti-osteoarthritic effects in a monosodium iodoacetate (MIA)-induced OA rat model. SGRE dose-dependently decreased nitric oxide (NO) production in LPS-induced RAW264.7 cells. Moreover, SGRE reduced the pro-inflammatory mediator (cyclooxygenase-2 (COX2), inducible NO synthase (iNOS), and prostaglandin E2 (PGE2)) and pro-inflammatory cytokine (interleukin-(IL)-1β, IL-6, and tumour necrosis factor (TNF-α)) levels. SGRE suppressed nuclear factor kappa B (NF-κB) and mitogen-activated protein kinase (MAPK) pathway activation in RAW264.7 macrophages, thus reducing inflammation. Rats were orally administered SGRE (150 or 200 mg/kg) or the positive control drug JOINS (20 mg/kg) 3 days before MIA injection, and once daily for 21 days thereafter. SGRE elevated the hind paw weight-bearing distribution, thus relieving pain. It also reduced inflammation by inhibiting inflammatory mediator (iNOS, COX-2, 5-LOX, PGE2, and LTB4) and cytokine (IL-1β, IL-6, and TNF-α) expression, downregulating cartilage-degrading enzymes, such as MMP-1, -2, -9, and -13. SGRE significantly reduced the SOX9 and extracellular matrix component (ACAN and COL2A1) levels. Therefore, SGRE is a potential therapeutic active agent against inflammation and OA.

## 1. Introduction

Osteoarthritis (OA) is a chronic disease of the articulating joints, characterised by the gradual degradation of articular cartilage, synovial inflammation, and remodelling of the subchondral bone [1]. Joint pain is a typical OA symptom, which severely affects the quality of life of patients, affecting both physical function and psychological parameters [2]. In addition, inflammation signalling pathways have been strongly implicated in the pathogenesis of OA [3,4]. Several studies have revealed that inflammation not only contributes to the initiation and progression of OA, but also mediates the development of pain [5]. With macrophages infiltrating the synovial membranes and activated macrophages being mediated through nuclear factor kappa B (NF-κB), c-Jun N-terminal kinase (JNK), phosphoinositide 3-kinase (PI3K), protein kinase B (Akt), the mechanistic target of rapamycin (mTOR), and other signalling pathways, inflammation considerably influences OA [6]. Increased pro-inflammatory cytokine levels in blood and synovial fluid in vivo, as well as an elevated inflammatory mediator production, lead to the expression of matrix metalloproteinases (MMPs) and other protein hydrolases, resulting in cartilage fracture [7,8]. Recent studies have suggested that the levels of conventional inflammatory mediators, such as tumour necrosis factor-alpha (TNF-α), interleukin (IL)-6 and IL-1β, nuclear factor kappa B (NF-κB), and cyclooxygenase 2 (COX-2), are increased in the synovial fluid and joint tissues of patients with OA [9,10,11]. Secreted inflammatory factors, such as TNF-α and IL-1β, which are regarded as major players among the pro-inflammatory cytokines, are critical mediators of the disturbed metabolism and enhanced catabolism of joint tissues observed in patients with OA [12]. NF-κB induces catabolic gene expression and promotes the expression of major pro-inflammatory and destructive mediators in OA, including cyclooxygenase 2 (COX2), inducible nitric oxide synthase (iNOS), and prostaglandin E2 (PGE2) [13,14]. Therefore, pharmacological treatments for the clinical management of OA mainly aim to relieve pain and inflammation [15,16].

The currently available therapies for the management of OA include nonsteroidal anti-inflammatory drugs (NSAIDs), which only provide temporary relief from clinical symptoms rather than repairing the tissue lesions or suppressing the progression of OA. Thus, identifying an effective and safe therapeutic strategy is urgently needed because NSAIDs are not very effective in relieving the pain associated with OA and exert adverse effects, such as bleeding and gastric ulceration, when used for a long period [17,18,19]. Existing evidence suggests that several natural products exhibit beneficial efficacy and are safe for use in the prevention and treatment of OA [20]. Therefore, we sought to determine the underlying mechanism of a natural product with excellent anti-inflammatory efficacy, which is the common factor of most OA drugs, and applied it to an OA animal model to demonstrate its efficacy. Thus, we investigated the anti-inflammatory properties of natural products using the RAW264.7 murine macrophage cell line, which has been widely used for in vitro inflammation studies [21].

*Siraitia grosvenorii* (Swingle) fruit (monk fruit or nahangwa in Korean, NHG), a member of the family Cucurbitaceae, has been used as a traditional Chinese medicine to treat asthma, acute gastritis, cough, constipation, sore throat, dire thirst, and bronchitis [22]. The extract of *S. grosvenorii* fruit, which contains sweet glycosides and is naturally low in calories, is commercially used as a sweetener [23]. Previous reports have shown that *S. grosvenorii* extract and its components have various biological activities, such as anti-oxidative, anti-tussive, anti-inflammatory, hypoglycaemic, immunologic, anti-carcinogenic, and hepatoprotective activities [22]. Since the *S. grosvenorii* fruit is extracted using water and then its residue is generally discarded, the *S. grosvenorii* residual extract (SGRE) is a novel low-cost material. We had previously demonstrated that SGRE exerts anti-asthmatic effects [24] and has anti-allergic and -inflammatory properties in skin inflammation animal models [25]; however, the anti-inflammatory and anti-osteoarthritic effects of SGRE in vitro and in vivo have not been studied. Therefore, we investigated the anti-inflammatory effects of SGRE in lipopolysaccharide (LPS)-induced RAW264.7 macrophages and its anti-osteoarthritic effects in a monosodium iodoacetate (MIA)-induced OA rat model.

## 2. Materials and Methods

### 2.1. SGRE Preparation and Chemical Profiling of SGRE

The SGRE used in this study was provided by Dongkuk Pharmaceutical (Seoul, Republic of Korea), and the manufacturing process was as follows: The air-dried *S. grosvenorii* residue was extracted twice using 70% ethanol, filtered, and concentrated under reduced pressure. After mixing the food-grade maltodextrin, the mixture was dried to obtain a powdered sample. The sample was then ground uniformly and used for the experiments (batch No. LHGE-210417, Hunan, Huacheng Biotech. Inc., Changshang, China). Mogroside V (MV), a standard compound used for quality control, was purchased from ChemFaces (Wuhan, Hubei, China). For the quantitative analyses, calibration curves with high linearity (*r*^2^ > 0.999) were plotted for six different concentrations (1000, 250, 100, 50, 20, and 10 μg/mL). The chromatographic solvents used for the analysis were of LC-MS grade (J. T. Baker, Phillipsburg, NJ, USA).

### 2.2. Ultra-High-Performance Liquid Chromatography-Charged Aerosol Detector (UHPLC-CAD) Analysis of SGRE

The SGRE samples were analysed using a Dionex Ultimate 3000 ultra-high-performance liquid chromatography (UHPLC) system (Thermo Scientific, Bremen, Germany) equipped with an autosampler and a detector coupled to a charged aerosol detector (CAD, Corona Veo RS). Samples (10 µL) were separated using a Waters XBridge® C18 column (4.6 × 250 mm, 5 µm) at a flow rate of 1 mL/min and eluted using a linear gradient of two mobile phases containing 0.1% formic acid in acetonitrile (A) and water (B). The linear gradient elution was optimised as follows: 0 min, 90% B; 0–1 min, 90% B; 1–44 min, 90–40% B; 44–45 min, 100–0% B; 45–54 min, 0–0% B; 54–55 min, 0–90% B; and 60 min, back to 90% B. The CAD was operated in the following conditions: a nitrogen gas pressure of 40 psi, an evaporation temperature of 50 °C, a high noise filter, and a detector response of 100 pA. The Chromeleon data system software (version 7.2.8) was used for the data acquisition and mathematical calculations.

### 2.3. Cell Culture and Lipopolysaccharide-Induced Inflammation

The RAW264.7 mouse cell line was purchased from the American Type Culture Collection (ATCC, Manassas, VA, USA). Cells were maintained in Dulbecco’s Modified Eagle’s medium supplemented with 1% penicillin/streptomycin and 10% heat-inactivated foetal bovine serum and incubated at 5% CO_2_ and 37 °C. After pre-treatment with SGRE (100, 200, and 400 µg/mL) for 2 h, the cells were incubated with 0.5 µg/mL LPS for 24 h to stimulate inflammation.

### 2.4. Cell Viability Assay

The viability of RAW264.7 cells was determined using a 3-(4,5-dimethylthiazol-2-yl)-2,5-diphenyltetrazolium bromide (MTT) (Sigma-Aldrich Chemical Co., St. Louis, MO, USA) assay. Briefly, RAW264.7 cells were seeded at a concentration of 1 × 10^5^ cells/well on a 96-well plate for 24 h. The cells were treated with different concentrations of SGRE (100, 200, and 400 µg/mL) for 24 h, and MTT (5 mg/mL) was then added to each well and the cells were incubated for an additional 3 h. The medium was removed, and the crystals were dissolved in dimethyl sulfoxide (DMSO, 100 μL/well) and stained with crystal violet. Their absorbance was determined at 490 nm using microplate reader (Molecular Devices, San Jose, CA, USA).

### 2.5. Measurement of the Pro-Inflammatory Cytokine and Mediator Levels

The nitric oxide (NO) content in the supernatant of each well was determined using the Griess method according to the manufacturer’s instructions (Promega, Madison, WI, USA). The levels of the pro-inflammatory cytokines IL-6, IL-1β, TNF-α, and PGE2 were determined using commercial enzyme-linked immunosorbent assay (ELISA) kits from R&D Systems (Minneapolis, MN, USA), as instructed by the manufacturer.

### 2.6. Western Blotting

The cells were washed twice with phosphate-buffered saline (PBS) and lysed on ice using cell lysis buffer (Cell Signaling Technology, Danver, MA, USA) according to the manufacturer’s instructions. Then, the cells were collected and centrifuged to obtain the protein. The extracted proteins were determined using a DC protein assay and bovine serum albumin (Bio-Rad, Hercules, CA, USA). The total protein samples (15–30 µg) were separated using sodium dodecyl sulfate polyacrylamide gel electrophoresis (SDS-PAGE) and transferred to polyvinylidene fluoride membranes (GE Healthcare UK Ltd., Buckinghamshire, Germany). The protein expression was determined using primary antibodies (1:1000 dilution) against p-ERK, ERK, p-p38, p38, p-JNK, JNK, p-NF-κB, NF-κB (Cell Signaling), COX-2, iNOS (Abcam), and β-actin (Santa Cruz). After 1 h, the membranes were washed three times, and then incubated with horseradish peroxidase-linked secondary antibodies (1:2000 dilution) for 1 h. The bands were visualised using enhanced chemiluminescence detection reagent and Image Quant LAS 4000 (GE Healthcare Life Sciences, Seoul, Korea). The visualised target protein levels were normalised to that of β-actin. The relative band density obtained from the images was determined using Image J software (US National Institutes of Health, Bethesda, MD, USA).

### 2.7. Induction of MIA-Induced OA in Rats

Male Sprague Dawley rats (7 weeks old, 190–210 g of body weight) were purchased from Orient Bio (Seongnam, Korea). After acclimation for one week, the rats were randomly divided into five groups of seven animals each: (1) normal control group (injected with saline + treated with 0.5% carboxymethyl cellulose (CMC)); (2) MIA group (injected with MIA + treated with 0.5% CMC); (3) 150 mg/kg SGRE-treated group (injected with MIA + treated with 150 mg/kg of SGRE); (4) 200 mg/kg SGRE-treated group (injected with MIA + treated with 200 mg/kg of SGRE); and (5) positive control group (injected with MIA + treated with 20 mg/kg of JOINS^®^ (SK Chemical Co., Ltd., Seongnam, Republic of Korea)). Briefly, 3 mg of MIA in 50 μL of saline was injected into the intra-articular joint of the right knee of each rat, except for those of the normal control group, under anaesthesia with a mixture of ketamine (25 mg/0.5 mL) and xylazine (20 mg/0.2 mL). SGRE or JOINS^®^ (first ever natural drug formula sold in Korea) dissolved in 0.5% CMC was orally administered to rats once a day for 3 days before the MIA injection, and for 21 days thereafter, until the end of the experiment. All experiments involving animals were approved by the Institutional Animal Care and Use Committee of Daejeon University and conducted in accordance with the Guide for the Care and Use of Laboratory Animals published by the US National Institutes of Health.

### 2.8. Joint Pain Assessment

The hind paw weight-bearing distribution between the MIA injection site on the right knee joint and the control side (left knee joint) was evaluated as an index of joint pain in each OA model. The changes in the hind paw’s weight bearing were determined using an incapacitance meter (Bioseb Co., Pinellas Park, FL, USA) 0, 7, 14, and 21 days after the intra-articular MIA induction, as previously described [26]. The weight distribution ratio was calculated using the following equation:

$$weight\;distribution\;ratio=\frac{right\;weight}{right\;weight+left\;weight}\times 100$$

### 2.9. Serum Analysis

Blood samples were collected via cardiac puncture under anaesthesia 22 days after OA induction. Serum was obtained by centrifugation at 3000× *g* for 10 min at 4 °C and collected and stored at −70 °C until the time of the ELISA experiments. The levels of IL-1β, IL-6, TNF-α, MMP-2, MMP-9, 5-LOX, COX-2, LTB4, and PGE2 were measured using ELISA kits from R&D Systems, according to the manufacturer’s protocols.

### 2.10. Real-Time Polymerase Chain Reaction (RT-PCR) Analysis

Total RNA was obtained from the knee joint tissue using RNeasy Mini Kit (Qiagen, Hilden, Germany). The RNA quality and amount were evaluated using NanoDrop 2000 Spectrophotometer (Thermo Fisher Scientific, Waltham, MA, USA). RNA (1 µg) was reverse-transcribed into cDNA using an iScript cDNA synthesis kit (Bio-Rad), according to the manufacturer’s protocol.

RT-PCR was conducted using a PCR mixture containing 1 mol/L of each primer and SYBR green master mix (Applied Biosystems, Foster City, CA, USA) and an RT-PCR System (Applied Biosystems, Foster City, CA, USA). The primer sequences utilised are present in Table 1. Amplification was conducted at 95 °C for 10 min, followed by 40 cycles of denaturation for 15 s, and annealing/extension at 60 °C for 10 min. The relative expression of the selected genes was calculated using the comparative cycle threshold (Ct) method [27] and expressed as rates of abundance relative to *GAPDH*, the reference gene.

### 2.11. Histopathology Analysis

The knee joints were fixed in 10% formalin, decalcified using 20% formic acid, and embedded in paraffin. Each specimen was cut into 5 µm thick sections and stained with H&E or Safranin-O/fast green (Sigma-Aldrich Chemical Co., St. Louis, MO, USA). Histological changes were examined using a light microscope (BX51; Olympus, Tokyo, Japan) and imaged using a digital camera (DP70; Olympus).

### 2.12. Statistical Analysis

The results are expressed as the mean ± standard error of the mean and were analysed using one-way analysis of variance, followed by Dunnett’s test or unpaired Student’s *t*-test, for multiple comparisons and two-group comparisons, respectively. All analyses were performed using GraphPad Prism 7.0 (GraphPad Software, San Diego, CA, USA), and *p*-values < 0.05 were considered statistically significant.

## 3. Results

### 3.1. Quantification of the Mogroside V Level in SGRE Using UHPLC-CAD

**Figure 1:**
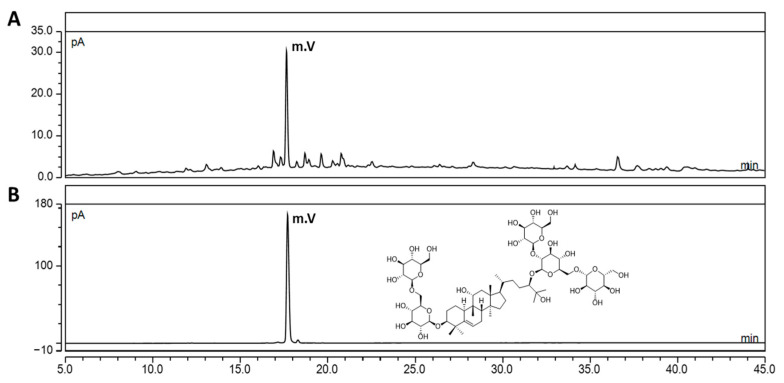
UHPLC-CAD chromatogram of SGRE (**A**) and MV of standard compound (**B**). Chemical structure of MV. The MV concentration was 1 mg/mL.

As shown in Figure 1, the complete chromatographic separation of the main component within SGRE, MV, was achieved within 45 min of monitoring the elution using CAD without interfering with the other components. Calibration curves were obtained by determining the peak area ratio of MV, which was fitted using least-squares linear regression calculations. The standard curve demonstrated a high correlation coefficient (*r*^2^ > 0.999) and was linear and reproducible. As a result of the quantitative analysis, the content of MV in SGRE was determined to be 5.3 mg/g per dry sample.

### 3.2. Effects of SGRE on the Viability of LPS-Induced RAW264.7 Macrophages

**Figure 2:**
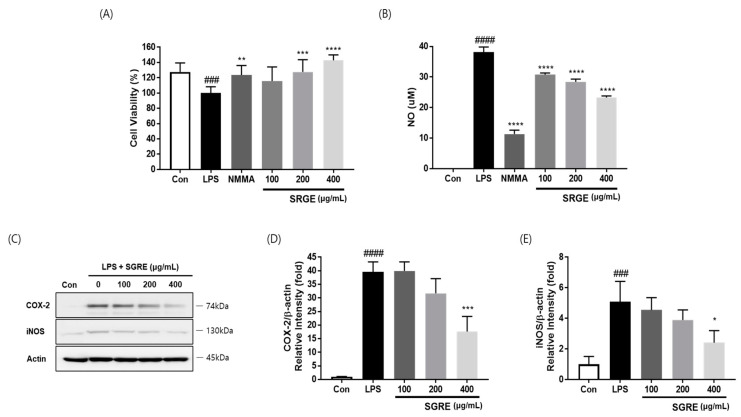
Effect of SGRE on NO production and COX-2 and iNOS expression in LPS-stimulated RAW264.7 macrophages. (**A**) Cell viability was measured using the MTT assay. (**B**) The culture supernatants were subsequently isolated and their NO levels were analysed. (**C**) The cells were lysed, and the lysates were analysed via immunoblotting using anti-COX-2 and anti-iNOS antibodies. (**D**,**E**) The intensity of the bands was quantified using Image J software. ^###^ *p* < 0.001; ^####^ *p* < 0.0001 vs. control cells. * *p* < 0.05; ** *p* < 0.01; *** *p* < 0.001; **** *p* < 0.0001 vs. LPS-treated cells.

The effect of SGRE on the cell viability of LPS-stimulated RAW264.7 macrophages was examined using a 3-(4,5-dimethylthiazol-2-yl)-2,5-diphenyltetrazolium bromide (MTT) assay. LPS (0.5 µg/mL) could significantly reduce the cell viability of RAW264.7 macrophages after 24 h of treatment. SGRE dose-dependently reversed this cell viability reduction (Figure 2A).

### 3.3. Effect of SGRE on NO Production and the Expression of COX-2 and iNOS in LPS-Induced RAW264.7 Macrophages

To measure the ability of SGRE to suppress inflammatory signals, we determined the NO production and COX-2 and iNOS expression in RAW264.7 cells stimulated with LPS. NG-Monomethyl-L-arginine mono-acetate salt (NMMA; L-NMMA, 50 μM), a nitric oxide synthase inhibitor, was used as a positive control. As shown in Figure 2, compared with that of the control, the NO production in LPS-stimulated RAW264.7 macrophages was significantly elevated. However, the SGRE treatment notably reduced the NO production in LPS-stimulated cells. Additionally, a remarkable elevation in the COX-2 and iNOS expression levels was observed in LPS-stimulated cells, compared with those of the control. The excessive COX-2 and iNOS expression induced by LPS was also profoundly alleviated by the treatment with SGRE in a concentration-dependent manner. Collectively, these results demonstrate that SGRE can inhibit the production of NO and the expression of COX-2 and iNOS.

### 3.4. Effect of SGRE on the Levels of Pro-Inflammatory Factors in LPS-Induced RAW264.7 Macrophages

**Figure 3:**
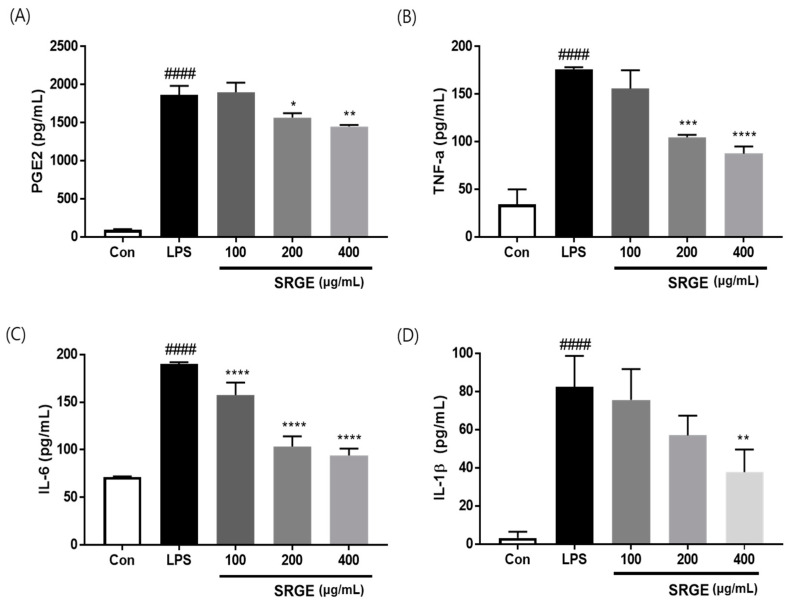
Effect of SGRE on inflammatory mediator and cytokine production in LPS-stimulated RAW264.7 macrophages. (**A**) PGE2, (**B**) TNF-α, (**C**) IL-6, and (**D**) IL-1β production in the culture media was quantified using ELISAs. ^####^ *p* < 0.0001 vs. control cells. * *p* < 0.05; ** *p* < 0.01; *** *p* < 0.001; **** *p* < 0.0001 vs. LPS-treated cells.

To assess the ability of SGRE to suppress inflammatory factor secretion, we investigated the inflammatory mediator and cytokine secretion in LPS-activated macrophages using ELISAs. As shown in Figure 3, the generation of pro-inflammatory cytokines, such as TNF-α, IL-6, IL-1β, and PGE2, was significantly elevated in LPS-activated macrophages. However, the treatment with 400 µg/mL SGRE dramatically reduced the expression of TNF-α, IL-6, and IL-1β by 62.4%, 81.0%, and 56.3%, respectively. In addition, at a dose of 200 to 400 µg/mL, SGRE remarkably suppressed the LPS-induced PGE2 production. Taken together, these data suggest that SGRE reduces the production of pro-inflammatory factors, thus preventing the progression of inflammation.

### 3.5. Effect of SGRE on MAPK Activation in LPS-Induced RAW264.7 Macrophages

**Figure 4:**
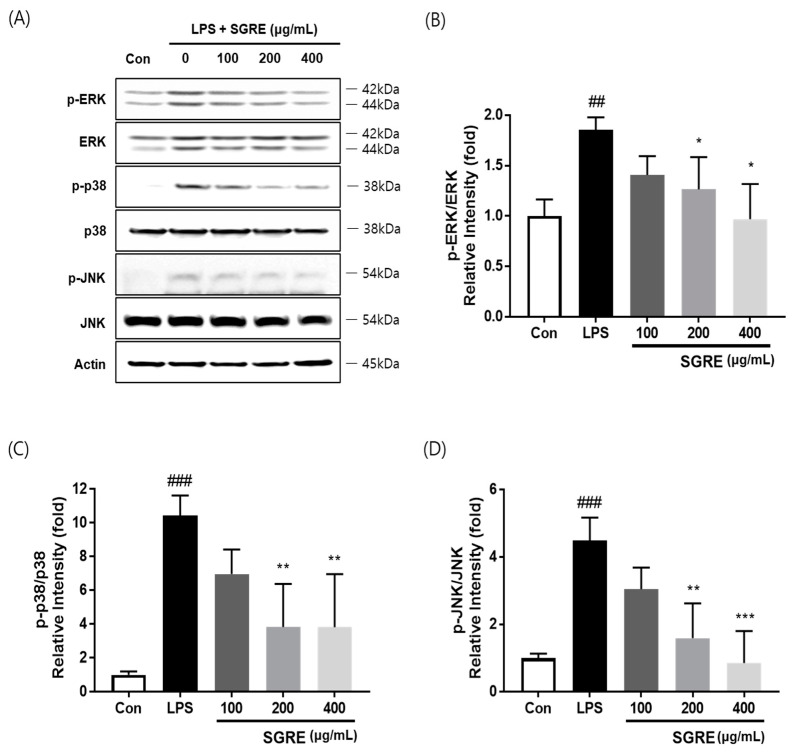
Effect of SGRE on the LPS-induced activation of the MAPK signalling pathway in RAW264.7 cells. (**A**) Representative Western blotting images of phosphorylated and total ERK, p38, and JNK. (**B**–**D**) Fold-change quantification of p-ERK (**B**), p-p38 MAPK (**C**), and p-JNK (**D**). ^##^ *p* < 0.01; ^###^ *p* < 0.001 vs. control cells. * *p* < 0.05; ** *p* < 0.01; *** *p* < 0.001 vs. LPS-treated cells.

The MAPK signalling pathway is a key regulator of LPS-induced inflammation [28]. Therefore, to investigate the effects of SGRE on the LPS-stimulated phosphorylation of p38, MAPK, ERK, and JNK in macrophages, we performed a Western blotting assay. As shown in Figure 4, LPS significantly induced the phosphorylation of p38, ERK, and JNK in macrophages. On the contrary, upon treatment with SGRE, the LPS-induced phosphorylation level of p38, ERK, and JNK decreased in a concentration-dependent manner. These results suggest that SGRE blocks p38, ERK, and JNK phosphorylation in the MAPK pathway, thus inhibiting inflammation in LPS-treated RAW 264.7 macrophages.

### 3.6. Effect of SGRE on IκB Degradation and NF-κB p65 Phosphorylation in LPS-Induced RAW264.7 Macrophages

**Figure 5:**
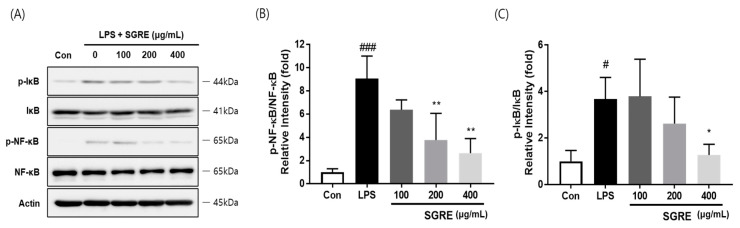
Effect of SGRE on the degradation or phosphorylation of proteins p65 and IκB in LPS-induced RAW264.7 macrophages. (**A**) Representative Western blotting images of phosphorylated and total p65 and IκB. (**B**,**C**) Quantification of the fold-change of phosphorylated p65 (**B**) and IκB (**C**). ^#^ *p* < 0.05; ^###^ *p* < 0.001 vs. control cells. * *p* < 0.05; ** *p* < 0.01 vs. LPS-treated cells.

The NF-κB pathway, activated by IκB, is a well-known regulator of the expression and secretion of pro-inflammatory enzymes and cytokines [29]. Therefore, to examine the effect of SGRE on the inhibition of NF-κB activity, the effects of SGRE on IkB phosphorylation were first investigated. As demonstrated in Figure 5A, SGRE suppressed the phosphorylation and degradation of IκB in a concentration-dependent manner in LPS-induced RAW264.7 cells. Furthermore, SGRE was confirmed to inhibit the phosphorylation of p65. These results show that SGRE induces an anti-inflammatory effect by inhibiting the activation of the NF-κB pathway in LPS-stimulated RAW246.7 cells.

### 3.7. Effects of SGRE on the Hind Paw Weight-Bearing Ratio

**Figure 6:**
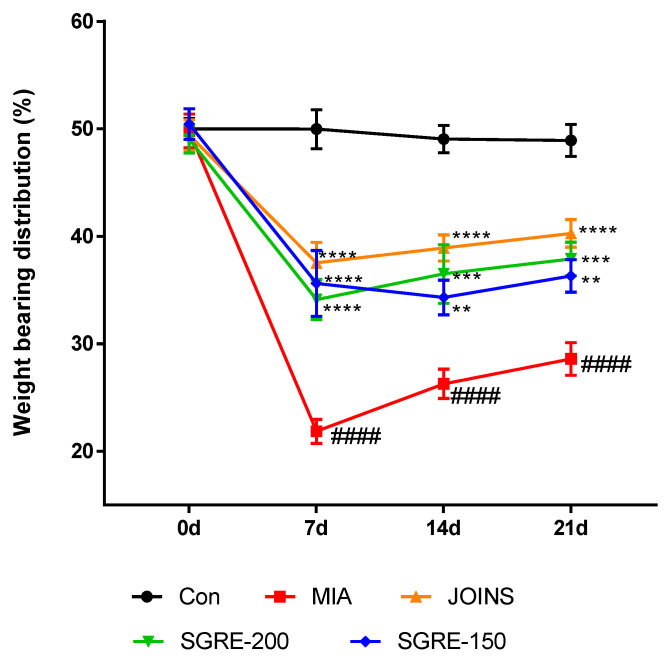
Effects of SGRE on the changes in hind paw weight-bearing distribution in MIA-induced OA rats. The results of the SGRE-treated groups were compared with those of the MIA-induced group. *n* = 7 per group. ^####^ *p* < 0.0001 vs. control group. ** *p* < 0.01; *** *p* < 0.001; **** *p* < 0.0001 vs. MIA-induced group.

Joint pain leads to a decreased weight-bearing distribution. A significant pain reduction was observed in both the positive control group and SGRE-treated group rats based on the increased hind paw weight-bearing ratios compared to those of the MIA group; therefore, the oral administration of SGRE at doses of 150 and 200 mg/kg significantly reduced joint pain in MIA-induced OA rats (Figure 6).

### 3.8. Effects of SGRE on the Mediators and Cytokines Involved in the Inflammatory Response

**Figure 7:**
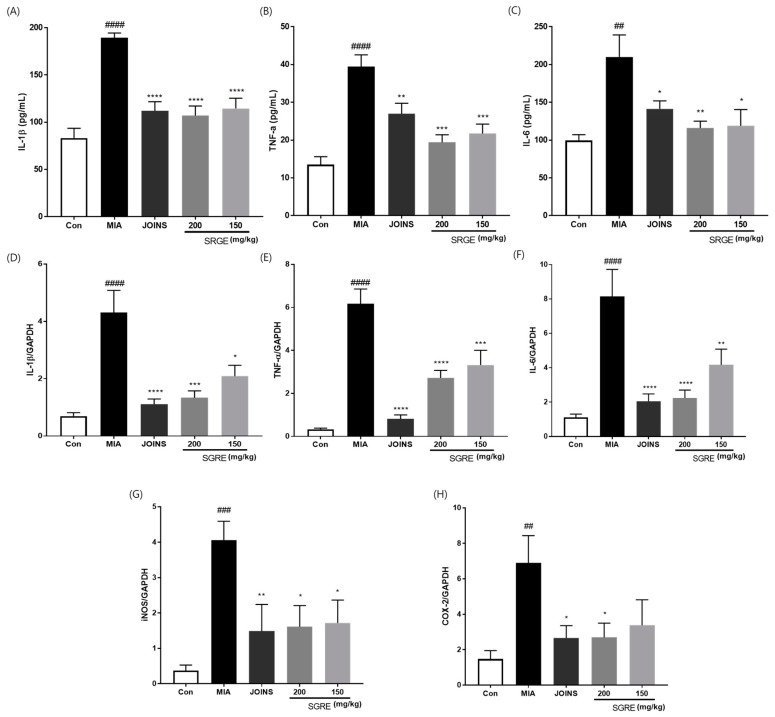
Effects of SGRE on the expression levels of inflammatory cytokines and enzymes in the MIA-induced group. The serum concentrations of (**A**) IL-1β, (**B**) TNF-α, and (**C**) IL-6. The mRNA levels of (**D**) IL-1β, (**E**) TNF-α, (**F**) IL-6, (**G**) iNOS, and (**H**) COX-2 in the joint articular cartilage tissue. *n* = 7 per group. ^##^ *p* < 0.01; ^###^ *p* < 0.001; ^####^ *p* < 0.0001 vs. control group. * *p* < 0.05; ** *p* < 0.01; *** *p* < 0.001; **** *p* < 0.0001 vs. MIA-induced group.

**Figure 8:**
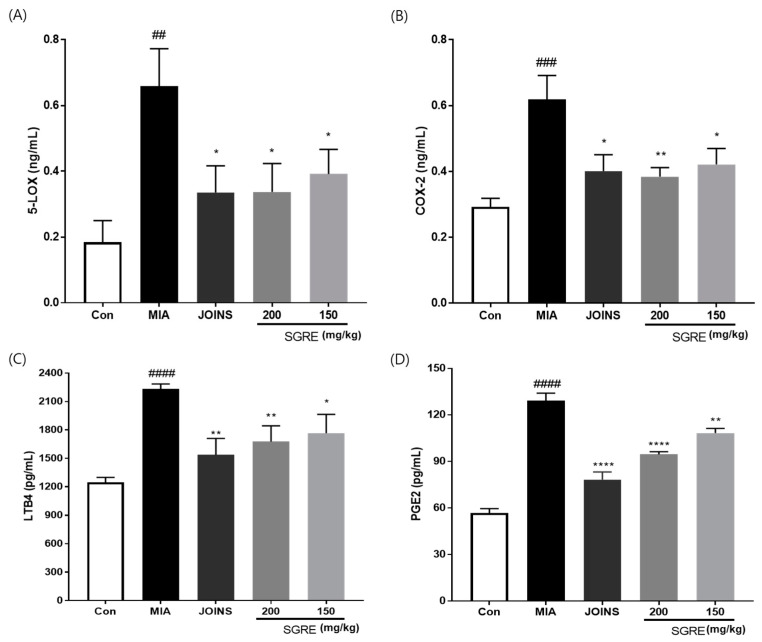
Effects of SGRE on the serum levels of inflammatory mediators in rats with MIA-induced OA. (**A**) Serum 5-LOX, (**B**) COX-2, (**C**) LTB4, and (**D**) PGE2 levels. *n* = 7 per group. *^##^ p* < 0.01; *^###^ p* < 0.001; *^####^ p* < 0.0001 vs. control group. * p < 0.05, ** p < 0.01; **** p < 0.0001 vs. MIA-induced group.

Since pro-inflammatory cytokines, such as IL-1β, TNF-α, and IL-6, are released during the inflammatory process, we investigated the anti-inflammatory effects of SGRE by measuring the cytokine levels in the serum and knee joint articular cartilage. The IL-1β, TNF-α, and IL-6 levels were significantly higher in the MIA group than in the control group. In contrast, the 150 and 200 mg/kg SGRE treatment significantly suppressed the production of IL-1β (63.41% and 70.68%), TNF-α (68.36% and 77.31%), and IL-6 (82.48% and 85.14%) in the serum, respectively (Figure 7A-C). Consistent with the results depicted in Figure 7D–F, SGRE significantly inhibited the mRNA expression of inflammatory cytokines (IL-1β, TNF-α, and IL-6) and inflammatory enzymes (COX-2 and iNOS) in a dose-dependent manner in MIA-induced OA rats. Furthermore, we investigated the effect of SGRE on the production of the inflammatory mediators LTB4, PGE2, 5-LOX, and COX-2. The serum levels of these inflammatory mediators were significantly lower in the SGRE-treated group than in the MIA group (Figure 8). Our data indicate that the treatment with SGRE reduced the secretion of LTB4 and PGE2 via the downregulation of both 5-LOX and COX-2. Therefore, these results suggest that SGRE exerts anti-inflammatory effects by modulating inflammatory enzymes, inflammatory mediators, and cytokines. 

### 3.9. Effects of SGRE on Cartilage Degradation in MIA-Induced Rats

**Figure 9:**
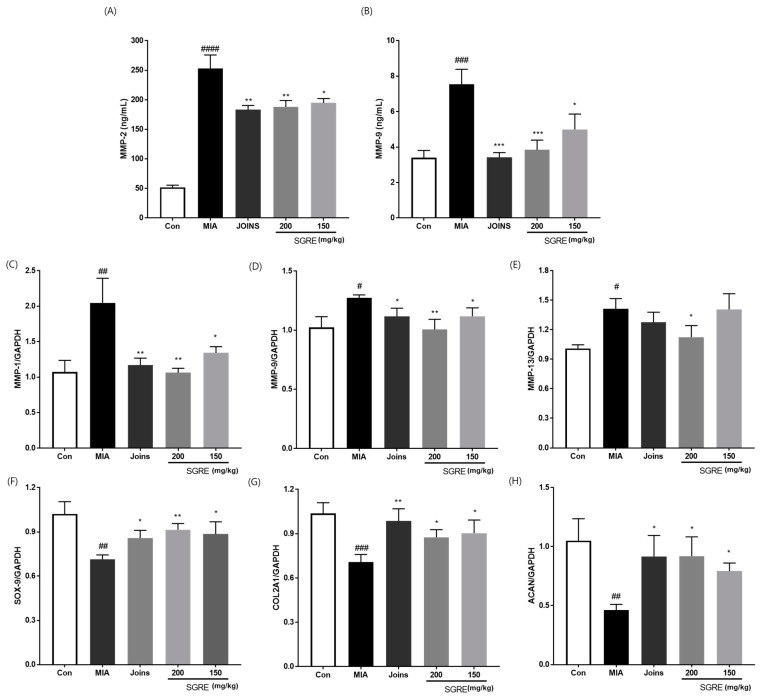
Effects of SGRE on cartilage degradation in rats with MIA-induced OA. The levels of (**A**) MMP-2 and (**B**) MMP-9 were determined using ELISAs. The mRNA expression levels of (**C**) MMP-1, (**D**) MMP-9, (**E**) MMP-13, (**F**) SOX-9, (**G**) COL2A1, and (**H**) ACAN were determined in the joint articular cartilage tissues using RT-PCR. *n* = 7 per group. *^#^ p* < 0.05; *^##^ p* < 0.01; *^###^ p* < 0.001; *^####^ p* < 0.0001 vs. control group. * p < 0.05; ** p < 0.01; *** p < 0.001 vs. MIA-induced group.

MMPs are proteolytic enzymes that regulate extracellular matrix (ECM) degradation and are involved in the progression of OA [30]. The expression of MMPs in the serum (MMP-2 and MMP-9) and knee joint articular cartilage (MMP-1, MMP-9, and MMP-13) was examined. The MMP-2 and MMP-9 levels notably increased in the MIA-induced group, whereas the 150 and 200 mg/kg SGRE-treated groups showed a significant decrease in MMP-2 and MMP-9 activity compared to that of the MIA-induced group. Similarly, the SGRE treatment significantly decreased the mRNA expression levels of MMP-1, MMP-9, and MMP-13 (Figure 9C-H). Conversely, the mRNA expression of ECM genes, such as *ACAN*, *SOX9*, and *COL2A1*, was dramatically reduced in the articular cartilage of MIA-induced rats. However, dose-dependent increases in ACAN, SOX9, and COL2A1 mRNA expression were observed in the 150 and 200 mg/kg SGRE-treated groups compared with those of the MIA-induced group.

**Figure 10:**
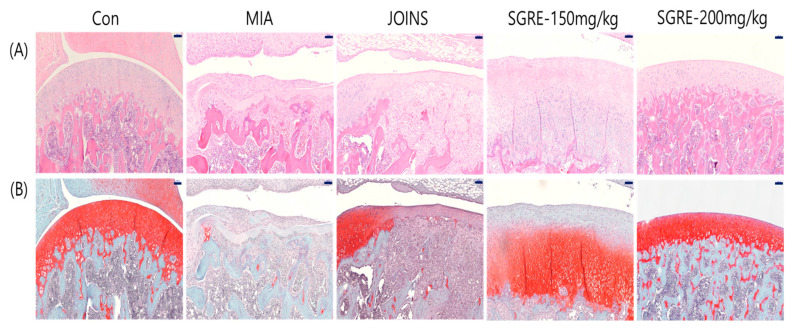
Histopathological features of the knee joint tissues of MIA-induced OA rats. The histopathological changes in the knee joint tissues were assessed using (**A**) H&E and (**B**) Safranin-O/Fast Green (scale bar = 100 µm).

Histopathological analysis using H&E staining showed cartilage degeneration, an irregular articular cartilage surface, and a greater increase in inflammatory cell infiltration in the synovial membrane and articular cartilage in the MIA-treated rat joints compared to those of the control group rat joints. In contrast, the SGRE-treated groups had reduced histological damage and cartilage degeneration, with a less irregular articular cartilage surface, and inflammatory cell infiltration (Figure 10A). In addition, we stained the proteoglycan layer and cartilage cells with Safranin-O to assess cartilage degradation. The red-stained control cartilage was destroyed by MIA, and the proteoglycan layer disappeared in the MIA-treated group. However, these histomorphological changes were reduced in the SGRE-treated rats (Figure 10B).

## 4. Discussion

OA is a slow progressive disease resulting in macrophage-related inflammation and articular cartilage degradation [31]. Macrophages, which are widely distributed inflammatory cells, play important roles in inflammation. When macrophages are exposed to LPS, which stimulates an inflammatory response, they activate the production of inflammatory cytokines, such as IL-1β, IL-6, and TNF-α, resulting in the release of COX-2 and iNOS, which catalyse the production of PGE2 and NO, which are inflammatory mediators [32]. NF-κB and MAPK signalling pathways both play central roles in mediating the LPS-stimulated inflammatory response [33,34]. MAPKs, such as ERK, JNK, and p38, are activated by phosphorylation and regulate inflammatory cytokine secretion via various stimuli [29,35,36,37]. The MAPK signalling pathways regulate the levels of transcription factors, such as NF-κB, that transcriptionally activate many genes involved in cell adhesion, survival, and immune response regulation [38]. Activation of the MAPK and NF-κB pathways initiates the transcription and expression of inflammatory genes, accumulating various mediators, such as TNF-α, IL-6, IL-1β, iNOS, and COX-2 [32,33]. Therefore, it is a key step in the treatment against inflammation, inhibiting the excessive production of inflammatory cytokines and mediators by suppressing the activation of NF-κB and MAPKs [39]. Our results reveal the anti-inflammatory mechanisms mediated by SGRE in the RAW264.7 macrophage cell line. LPS stimulation of RAW264.7 macrophages increased the activity of the NF-κB and MAPK signalling pathways; however, the SGRE treatment dramatically inhibited p-IκB and NF-κB-p65 activities and the phosphorylation of p38, MAPKs, JNK, and ERK. Furthermore, the present study showed that SGRE reduced the LPS-induced production of NO and ROS and the expression of IL-1β, IL-6, and TNF-α in RAW264.7 cells. Reduced NO levels and TNF-α, IL-6, and IL-1β secretion inhibit the expression of iNOS and COX-2, which are key enzymes for NO and PGE2 production in inflammatory conditions. Taken together, our results suggest that SGRE reduced the levels of inflammation-related markers, such as pro-inflammatory mediators, cytokines, and enzymes by lowering the NF-κB and MAPK signalling pathway activities.

Cartilage is composed of chondrocytes and ECM. SOX9 plays an important role in chondrocyte differentiation and chondrogenesis, and type II collagen (COL2A1) and proteoglycans such as aggrecan are major components of the ECM [40]. MMPs are enzymes responsible for ECM degradation and are expressed in the joint tissues of patients with OA. The gelatinases (MMP-2 and MMP-9) degrade gelatin, which is a collagen fragment produced by collagenases (MMP-1, MMP-8, and MMP-13), cartilage cells, such as fibronectin, elastin and laminin, various types of collagens, and components of ECM [41]. MMP-13 is responsible for the degradation of COL2A1, which is the main collagen type found in articular cartilage [42]. Moreover, inflammatory cytokines decrease the synthesis of key components of the ECM, such as proteoglycans, aggrecan, and COL2A1, and conversely, contribute to the increased synthesis and release of various proteolytic enzymes, including cartilage-degrading enzymes, such as MMPs and a disintegrin and metalloproteinase with thrombospondin motifs (ADAMTS) [43]. Particularly, IL-1β and TNF-α are mainly produced by macrophages, which drive the inflammatory and destructive responses in articular cartilage. Therefore, in order to prevent the progression of OA, it is important to control the inflammatory process that takes place inside the articular cartilage tissue and the resulting cartilage destruction. In the present study, we found that the IL-1β, IL-6, TNF-α, iNOS, and COX-2 levels in the serum and joint articular cartilage tissues were elevated in the MIA-induced OA model and that the SGRE treatment at 150 and 200 mg/kg significantly decreased the cytokine levels. Moreover, the SGRE-mediated suppression of 5-LOX and COX-2 led to a significant decrease in LTB4 and PGE2 expression. The inhibition of inflammation progression in SGRE-treated OA rats might therefore be attributed to the decrease in the levels of inflammatory mediators and cytokines. Additionally, the inhibition of inflammatory cytokines by SGRE resulted in decreased levels of the cartilage-degrading enzymes MMP-1, 2, 9, and 13, in the serum and articular cartilage tissues. SGRE increased the transcriptional activity of SOX9, which synthesises the two main components of the cartilage ECM, aggrecan (ACAN) and COL2A1; however, it also increased the expression of these components. These results suggest that SGRE reduced the expression of SOX9 and the ECM components ACAN and COL2A1 through its anti-inflammatory activity.

The hind paw weight distribution in the rat MIA-induced model of OA is predictive of the effects of analgesic and anti-inflammatory agents [44]. We demonstrated the recovery of the hind paw weight-bearing ability of SGRE-treated rats when compared with that of MIA-induced rats, indicating that SGRE may relieve joint pain. Similar to JOINS, a medicine that improves OA symptoms, SGRE treatment also relieved joint pain, which is a symptom of OA.

In our previous study, we reported that the SGRE extract contains mogroside V, mogroside IV, mogroside III, 11-oxo-mogroside V, and mogroside II [24]. MV, which is considered the main active ingredient of *S. grosvenorii* and has a wide range of biological activities, is known to have most pharmacological activities [45]. When analysing the compounds present in natural products, one of the biggest problems concerning liquid chromatography using a UV detector is the detection of analytes when the compounds lack chromophores. Before CAD became prevalent, low-wavelength UV detectors, refractive index detectors, and evaporative light scattering detectors were mainly used for the quantitative analysis of chromophore-free compounds [36]. Recently, a general-purpose detection method called charged aerosol detection, which exhibits a uniform analytical response capable of detecting all non-volatile and some semi-volatile compounds, regardless of their chemical structure, has been preferred [46]. In this study, to ensure the biological efficacy of SGRE as an anti-inflammatory and OA treatment material and maintain consistent quality control, its chemical composition was examined using UHPLC-CAD, the marker component MV, its main component, and a content standard of 5.3 mg/g.

## 5. Conclusions

The present study demonstrated, for the first time, that SGRE inhibited the inflammation induced by LPS via MAPKs and NF-κB signalling pathways in RAW264.7 cells. SGRE treatment also inhibited the inflammatory response by downregulating the inflammatory mediators (iNOS, COX-2, 5-LOX, PGE2, and LTB4) and cytokines (IL-1β, IL-6, and TNF-α). SGRE decreased the catabolism of the articular cartilage via the downregulation of ECM-degrading enzymes (MMPs) and increased the anabolism by upregulating SOX9 and ECM synthesis-related genes (*ACAN* and *COL2A1*). Collectively, our findings suggest that SGRE could be useful for the prevention and treatment of inflammation and its related diseases. Based on the results of this preclinical animal study, we are now progressing to a clinical study of SGRE on patients with OA.

## Figures and Tables

**Table 1:** Primer sequences used for the mRNA expression analyses.

Gene		Primer Sequence
*IL-1β*	Forward	5′-ACAAGGCTGCCCCGACTAT-3′
	Reverse	5′-CTCCTGGTATGAAGTGGCAAATC-3′
*IL-6*	Forward	5′-GCCCTTCAGGAACAGCTATGA-3′
	Reverse	5′-TGTCAACAACATCAGTCCCAAGA-3′
*TNF-α*	Forward	5′-ACAAGG CTGCCCCGACTAT-3′
	Reverse	5′-CTCCTGGTATGAAGTGGCAAATC-3′
*MMP-1*	Forward	5′-CTCCCTTGGACTCACTCATTCTA-3′
	Reverse	5′-AGAACATCACCTCTCCCCTAAAC-3′
*MMP-9*	Forward	5′-GCGCCAGCCGACTTATGT-3′
	Reverse	5′-AATCCTCTGCCAGCTGTGTGT-3′
*MMP-13*	Forward	5′-ACGTTCAAGGAATCCAGTCTCTCT-3′
	Reverse	5′-GGATAGGGCTGGGTCACACTT-3′
*SOX-9*	Forward	5′-CTGAAGGGCTACGACTGGAC-3′
	Reverse	5′-TACTGGTCTGCCAGCTTCCT-3′
*COL2A1*	Forward	5′-GCAACAGCAGGTTCACGTACA-3′
	Reverse	5′-TCGGTACTCGATGATGGTCTTG-3′
*ACAN*	Forward	5′-GAAGTGGCGTCCAAACCAA-3′
	Reverse	5′-CGTTCCATTCACCCCTCTCA-3′
*GAPDH*	Forward	5′-TGGCCTCCAAGGAGTAAGAAAC-3′
	Reverse	5′-CAGCAACTGAGGGCCTCTCT-3′

## Data Availability

The data are contained within the article.
